# Long-term prognosis of 35 patients with methionine adenosyltransferase deficiency based on newborn screening in China

**DOI:** 10.3389/fcell.2022.1059680

**Published:** 2023-01-10

**Authors:** Fan Tong, Yuchen Zhang, Chi Chen, Ling Zhu, Yijun Lu, Zhanming Zhang, Ting Chen, Jiaxuan Yan, Jing Zheng, Xiaoxu Zhao, Duo Zhou, Xin Yang, Rulai Yang, Xiaohui Cang, Pingping Jiang, Qiang Shu

**Affiliations:** ^1^ Department of Genetics and Metabolism, The Children’s Hospital, National Clinical Research Center for Child Health, Zhejiang University School of Medicine, Hangzhou, China; ^2^ Institute of Genetics, Zhejiang University School of Medicine, Hangzhou, China; ^3^ Department of Molecular Genetics, University of Toronto, Toronto, ON, Canada

**Keywords:** methionine adenosyltransferase deficiency (MATD), long-term prognosis, MAT1A, neurological deficits, hypermethioninemia, S-adenosylmethionine

## Abstract

Methionine adenosyltransferase deficiency (MATD) is a rare metabolic disorder caused by mono- or biallelic *MAT1A* mutations that are not yet well understood. Of the 4,065,644 neonates screened between November 2010 and December 2021, 35 individuals have been diagnosed with an estimated incidence of 1: 116,161 by a cutoff value of methionine 82.7 μmol/L and follow-up over 11 years. MATD patients with autosomal recessive (AR) type had higher clinical and genetic heterogeneity than those with autosomal dominant (AD) type. Fifteen unrelated AD patients harbored one well-known dominant variant, c.791 G>A or c.776 C>T, and were clinically unaffected with a mean plasma methionine (Met) value <300 μmol/L. Twenty AR cases have unique genotypes and presented a wide range of clinical abnormalities from asymptomatic to white matter lesions. Of them, 10 AR patients displayed severe manifestations, such as verbal difficulty, motor delay, development delay, and white matter lesions, with mean Met >500 μmol/L and thereby were treated with a methionine-restricted diet alone or in combination with betaine, folate, or vitamin B6, and were healthy finally. Neurological abnormalities were evidenced in two patients (P16 and P27) with Met values >800 μmol/L by MRI scan. Neurological abnormalities were reversed here by liver transplantation or by the determination of S-adenosylmethionine supplementation. Additionally, 38 variants of *MAT1A* were distributed within patients and carriers, of which 24 were novel and mostly predicted to be damaged. Our findings with an extensive clinical and genetic dataset provided new insights into its diagnosis and treatment and will be helpful for its optimal management in the future.

## Introduction

Methionine adenosyltransferase deficiency (MATD, #250850) is a rare metabolic disorder resulting in methionine adenosyltransferase (MAT) deficiency, with isolated hypermethioninemia and a low level of hepatic S-adenosylmethionine (SAMe). MATD can be transmitted either as autosomal dominant (AD) or autosomal recessive (AR) traits in which mono- or biallelic *MAT1A* variants have been identified, respectively (Mudd et al., 2003; Mudd, 2011). Although the majority of patients are described as mild or asymptomatic, some cases present with malodorous breath (Furujo et al., 2012), acrodermatitis enteropathica (Nashabat et al., 2018), or even neurological deficits (Chamberlin et al., 2000; Braverman et al., 2005; Kido et al., 2019). To date, MATD has been recommended as a secondary condition in the Recommended Uniform Screening Panel (RUSP). However, it is still unclear whether MATD is a benign genetic disorder. Chien and colleagues found that half of their samples of MATD patients developed neurological symptoms later in life (Chien et al., 2015). To advance the understanding of MAT deficiency, scientists and physicians have promoted diagnostic strategies, delineated manifestations and genotypes, and evaluated treatments for MATD worldwide (Barić et al., 2017). Nevertheless, the long-term prognosis of MATD, as well as clinical and genetic characteristics, is still limited and incompletely understood. Based on a total of 4,065,644 neonates screened on the NBS program between November 2010 and December 2021, 35 patients with MATD were retrospectively analyzed for clinical symptoms, genotypes, treatment and outcomes of MATD to provide new insight to improve the understanding of MATD and optimal management.

## Materials and methods

### Newborn screening and subjects

All patients with a diagnosis of MATD and carriers based on newborn screening (NBS) program, genetic testing, and follow-up between November 2010 and December 2021 in Zhejiang Neonatal Screening Center, in which a total of 4,065,644 neonates were screened covering 364 maternity units in 90 counties. All procedures followed were in accordance with approval from the Research Ethics Committees, the Children’s Hospital of Zhejiang University School of Medicine. Patient information was tabulated without individual identifiers.

Tandem mass spectrometry (MS/MS) was used to detect the concentrations of amino acids and acylcarnitine with dry-spot-blood samples in our NBS center. The reference value of methionine (Met) is 7.18–41.35 μmol/L and the reference range of Phenylalanine (Phe) was 23.3–100 μmol/L. Based on NBS screening data, the cut-off value for Met was set to 82.7 μmol/L, an upper limit of normal values in the 99.9th centile healthy newborns. The cut-off value for the ratio of Met/Phe was 1.1. Plasma total homocysteine (tHcy) was considered to be elevated at a concentration above 15 μmol/L. Newborns were recalled due to a higher Met concentration of more than 82.7 μmol/L alone or due to a higher ratio of Met/Phe. If the second test was also positive (Met >82.7 μmol/L or Met/Phe >1.1), the newborn was referred for a gene test. Cases associated with cystathionine β-synthase deficiency, tyrosinemia type I, or severe liver diseases were excluded from this study. No other positive results of blood physiological indicators for IEME were detected in MATD patients.

### Genetic testing and analysis

Next-generation sequencing was carried out by BGI (Shenzheng, China) or by MyGenostics (Beijing, China) during 2010–2015. Other samples were analyzed with a targeted gene panel by next-generation sequencing (NGS) in our Genetic Diagnostic Laboratory at the Children’s Hospital as previously described (Lin et al., 2020) and contained 166 genes involved in an inborn error of metabolism (IEMD, including causative genes associated with MATD (*MAT1A, GNMT, and AHCY*) and Met-related IEMD, such as homocystinuria (*CBS, MTHFR, ABCD4, LMBRD1, MMACHC, MMADHC, CD320, MLYCD,* etc.). Sanger sequencing was performed to verify variants detected in the panel. Novel missense variants were evaluated with several automatic tools (SIFT, PolyPhen-2, MutationTaster, CADD, etc.). Variants leading to truncated protein were classified as pathogenic (P) or likely pathogenic (LP) according to the recommendations of the American College of Medical Genetics and Genomics (ACMG) (Richards et al., 2015).

### Procedure of follow-up, clinical data collection, and intervention

All positive cases were followed up by specialists at the Department of Genetics and Metabolism. Regular intervals ranged from every 1–3 months, depending on the severity of manifestations, and were followed for cases at 1 year and every 3–6 months for cases beyond 1 year. Data from profiles of serum amino acids (including methionine), tHcy, and routine blood tests for the functions of the liver and kidney were monitored regularly during the follow-up. Other indicators, such as blood gases and electrolytes, serum glucose, and lactic acid, were collected irregularly. Bayley Scales of Infant Development, or the Ages-Stages Questionnaire (ASQ) or Gesell Developmental Schedule were used to assess the developmental status of MATD patients. Length/height-for-age and weight-for-age standards were according to China growth standards (0–3 years old) and WHO Child Growth Standards (>3 years old). Some special patients with severe clinical abnormalities or with extreme elevation of plasma Met (>800 μmol/L) were referred to undergo a brain MRI (magnetic resonance imaging) scan. If suspected cases harbored an unreported variant of *MAT1A*, the Met value and the ratio of Met/Phe from one parent carrying the same variant were detected.

Interventions were considered when plasma Met was >500 µmol/Lin infants. A low-methionine formula is usually used. Vitamin B6 and betaine were considered when tHcy increased slightly twice or exceeded 30 μmol/L. One patient was given oral SAMe. One patient underwent liver transplantation.

### Statistical analysis

Met values were illustrated using GraphPad Prism 8.0. A 2-tail paired or unpaired Student’s *t*-test or ordinary one-way ANOVA was performed for comparisons. *p* < .05 was considered significant.

## Results

**FIGURE 1:**
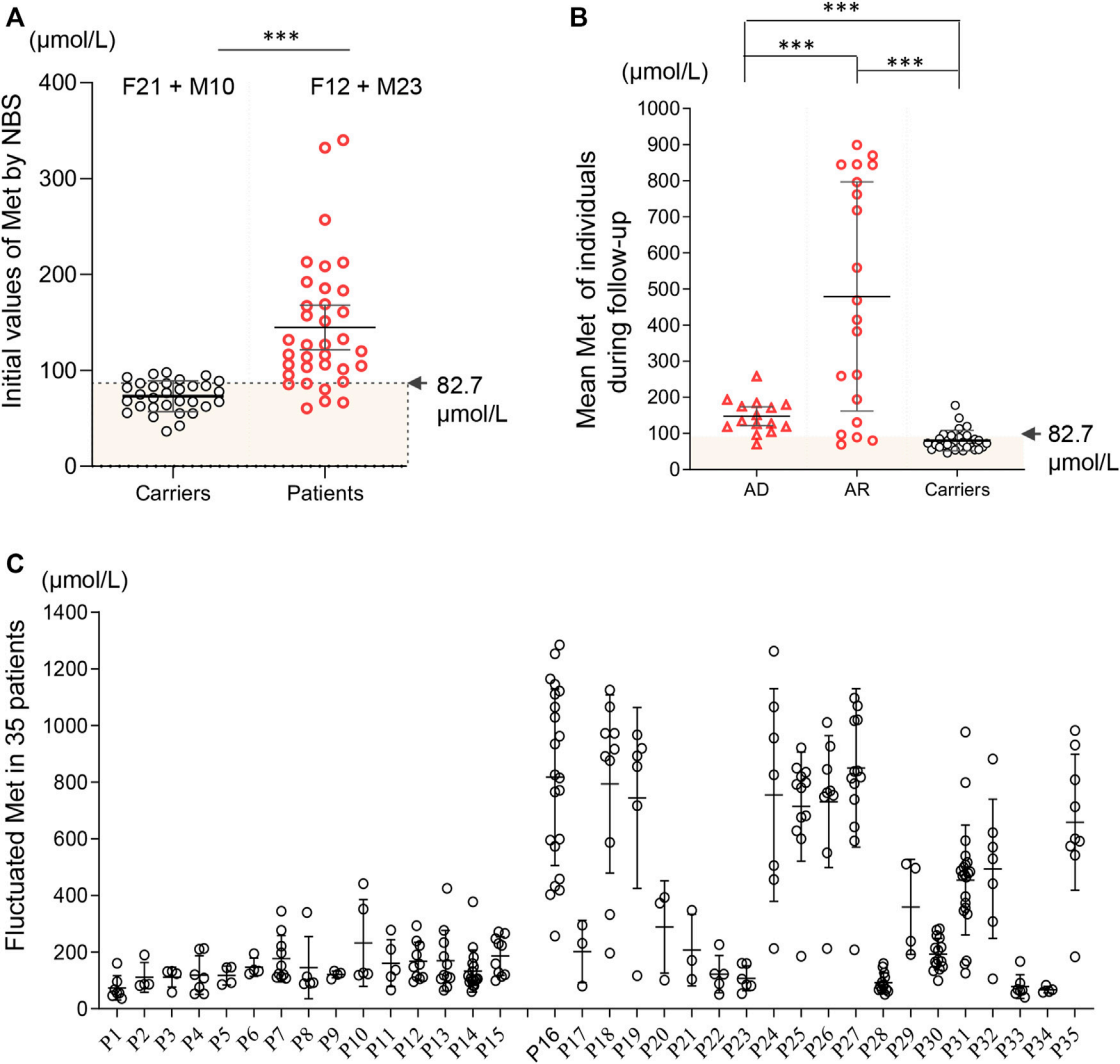
Methionine values based on newborn screening and follow-up among 35 patients and 31 carriers. **(A)** Initial Met values by newborn screening distributed in carriers (black) and patients (red). Significant difference was found in the mean initial Met values between carriers (73 μmol/L) and patients (145 μmol/L). F12 + M10 = 12 females and 10 males. 4 asymptomatic patients (AD-P11; AR-P17, P33, P34) had initial Met <82.7 μmol/L. **(B)** Mean plasma Met during follow-up in patients (AD, AR) and carriers. Asymptomatic patients P1, P33 and P34 had a mean Met below 82.7 μmol/L. **(C)** Fluctuated plasma Met values (including minimum, maximum and mean value of Met) during follow-up among each 35 individuals. *, *p* < .05; **, *p* < .01; ***, *p* < .001.

A total of 4,065,644 neonates were screened and 35 positive cases were diagnosed with MATD, which led to an estimated incidence of 1/116,161 in the MATD cohort of Zhejiang Province, a comparable incidence to that reported in Japanese (Nagao et al., 2013). Simultaneously, 31 carriers were identified and underwent follow-up who harbored novel heterozygous variants. A statistically significant difference existed in the initial Met concentrations between carriers and patients (*p* < .001, Figure 1A), of which patients ranged from 60 to 332 μmol/L with a mean value of 145 μmol/L, while carriers ranged from 42 to 98 μmol/L with a mean value of 73 μmol/L. Compared to AD patients, AR patients have more severe phenotypes, such as development delay, verbal difficulty, motor delay, and white matter lesions, and harbor various genotypes (Table 1). All reported variants in MATD were reviewed and are summarized in Supplementary Table S1.

**TABLE 1:** Clinical features and genotypes in 35 MATD cases.

Cases/sex	NBS			Allele1	Allele2	Follow-up		Clinical symotom	Treatment
	Met (µmol/L)	Met/Phe	tHcy (µmol/L)			Mean (min-max) met (µmol/L)	Min-max met/Phe		
P1, M	95	1.26	11.6	c.776C>T/p.A259V		70.1 (36–161)	0.9–1.55		—
P2, F	86	1.28	9.2	c.776C>T/p.A259V		119.1 (82–190)	1.13–2.89		—
P3, M	132	1.13	13.2	c.776C>T/p.A259V		104.8 (59–132)	1.34–3.63		—
P4, F	120	1.85	7.9	c.776C>T/p.A259V		118.6 (53–213)	0.92–4.88		—
P5, M	85	0.92	12.7	c.791G>A/p.R264H		127.8 (92–147)	2.53–3.29		—
P6, M	133	2.21	13.7	c.791G>A/p.R264H		151.4 (123–194)	2.67–5.01		—
P7, M	151	2.68	16.1	c.791G>A/p.R264H		179.5 (107–345)	1.82–5.46	serum zinc↓ at 2y	
P8, F	339	3.02	18.3	c.791G>A/p.R264H		96 (89–113)	2.7–2.9		—
P9, F	105	1.75	NA	c.791G>A/p.R264H		126.6 (121–133)	1.56–3.12		lost
P10, F	126	2.51	13.2	c.791G>A/p.R264H		258.7 (118–441)	2.72–12.07	serum zinc↓ at 6 m	
P11, F	67	1.25	16.2	c.791G>A/p.R264H		184.6 (114–278)	3.3–9.6		—
P12, F	106	2.14	14.2	c.791G>A/p.R264H		174.6 (96–294)	0.27–6.65		
P13, F	157	2.71	18.1	c.791G>A/p.R264H		170.9 (66–425)	1.45–9.69		
P14, F	116	1.4	14.4	c.791G>A/p.R264H		134.3 (61–377)	1.21–5.09		—
P15, M	114	2.21	19.3	c.791G>A/p.R264H		193.9 (99–271)	2.69–6.23		—
P16^*^, M	257	4.58	59	**c.695C>T/p.P232L**		844.1 (404–**1283)**	5.12–**29.14**	White matter lesions	Liver transplantation
P17, M	80	1.47	20.2	c.188G>T/p.G63V	c.361G>A/p.V121I	262.9 (230–295)	5.4–6.73		—
P18, F	332	5.86	27.1	c.656G>C/p.R219P	c.895C>T/p.R299C	844.6 (196**–1125)**	1.63–30.5	Transient jaundice	Met-restricted diet
P19, M	117	2.15	17	c. 765_768delCCAG/p.P255Pfs*35	c.274T>C/p.Y92H	869.9 (854–966)	19.88–28.8		Met-restricted diet
P20, M	101	1.96	17.5	c.181A>C/p.K61Q	c.943 C>T/p.L315F	382.6 (372–392)	5.63–6.18	Transient jaundice	—
P21, M	103	1.57	19.8	c.422A>G/p.Y141C	c.755T>C/p.I252T	259.2 (170–348)	4.57–8.27		—
P22, M	88	1.19	16.1	c.1070C>T/p. R357L	c.1070C>T/p. R357L	130.6 (50–227)	1.32–5.08		—
P23, M	161	2.82	17	c.895C>T/p. R299C	c.580G>A/p.A194T	96.7 (54–160)	0.98–5.11		—
P24, M	212	4.3	42	c.292G>A/p.G98S	c.895C>T/p. R299C	845.1 (456**–1262)**	15.6–39.56	ALP↑ at 6 m	Met-restricted diet, B6
P25, F	186	3.38	27	c. 875G>T/p.R292L	c. 875G>T/p.R292L	761.9 (599–921)	16.2–26.7	Verbal difficulty at 3y	Met-restricted diet, folate, betaine
P26, M	213	12.65	16.3	c.350T>C/p.I117T	c.188G>T/p.G63V	795.9 (550–**1011)**	24.39–30.4	transient motor delay, Bone age advanced 1 year	Met-restricted diet
P27^*^, M	209	3.98	20.4	c.769G>A/p.G257R	c.769G>A/p.G257R	899.1 (592–**1408)**	11.28–44.7	White matter lesions	Met-restricted diet, B6, SAMe
P28, M	127	3.19	18.2	c.478C>T:p.L160F	c.188G>T/p.G63V	89.4 (51–160)	1.08–4.59		—
P29, M	192	4.04	22.1	c.188G>T/p.G63V	c. 964A>G:p.I322V	415 (238–511)	3.45–13.36		—
P30, M	167	2.48	22.2	c.653T>C, p. M218T	c.836C>T, p. G279W	194.5 (99–282)	2.51–6.89		—
P31, M	169	3.15	41.5	c.769G>A/p.G257R	c.875G>T p.R299C	468.9 (159–977)	3.58–17.74	Developmental quotient 68 at 2y	Met-restricted diet, B6, taurine
P32, M	106	2.67	35	c.895C>G/p.R299G	c.38dupT/p.L13Lfs*15	558.8 (308–881)	7.16–33.82		Met-restricted diet, betanie
P33, M	68	1.11	13	c.188G>T/p.G63V	c.242G>A/p.R81Q	80.1 (41–167)	1.08–2.32		—
P34^#^, F	60	0.61	8.5	c.547C>G/p.Q183E	c.242G>A/p.R81Q	69.7 (58–83)	0.94–1.08		—
P35^†^, M	183	5.84	32	695C>T, p.P232L	c.608T>A p.I203N; c.922G>C p.A308P	718 (543–983)	14.6–33.0		Met-restricted diet, betaine

P16^*^, Met values here were recorded before liver transplantation; P34^#^, Normal biochemical indexes; P35^†^, Carrying 3 variants of *MAT1A*; “—“, no treatment applied. 2 years = 2-year-old, 6 m = 6-month-old; F, female; M, male.

### Patients with autosomal dominant MATD

There were 15 unrelated MATD patients (6 male and 9 female patients) diagnosed with a monoallelic variant of *MAT1A*, of which 11 individuals (P5-P15) carried c.791G>A/p. R264H and four individuals carried c.776C>T/p. A259V. Patient 1 (P1) and patient 9 (P9) were born prematurely, while their mothers’ pregnancies were uneventful to that point. Consistent with previous reports (Couce et al., 2008; Martins et al., 2012; Couce et al., 2013), variants c.791G>A and c.776C>T are common in the AD type, with frequencies of 73% (11/15) and 27% (4/15) respectively. Patients harboring c. 776C>T had an initial Met range of 86–132 μmol/L, with a Met/Phe ratio of 1.13–1.85 and normal tHcy. Patients harboring c.791G>A had an initial Met range of 67–339 μmol/L, with a Met/Phe ratio of .92–3.02. However, slight elevations in plasma tHcy were detected among five patients harboring c.791G>A. Patients 1–15 were followed up for 4–111 months with Met levels fluctuating from 36 to 441 μmol/L without significant clinical symptoms. Each of them had normal growth and development without drug treatment during follow-up, while a transient decrease in serum zinc was observed in P7 at 2 years old and P10 at 6 months old. The mean initial Met of these 15 patients was 129 μmol/L, and the mean plasma Met during follow-up was 147 μmol/L (Figure 1B). All AD patients were healthy and had no clinical sequelae.

### Patients with autosomal recessive MATD

Twenty cases (17 male and 3 female patients) of AR-type MATD were diagnosed with persistently high plasma Met concentrations and compound heterozygous or homozygous variants of *MAT1A* without other pathogenic genes in this metabolic disorder. All patients were full-term infants. Compared to AD patients, AR patients have more complicated phenotypes and genotypes. The initial Met concentrations in 20 AR cases ranged from 60 to 332 μmol/L, with a mean value of 157 μmol/L (Table 1). No significant difference was exhibited in the initial Met concentrations between the AD and AR groups. However, the plasma Met values, during follow-up, had a wider range from 41 to 1408 μmol/L with a mean value of 479 μmol/L (Figure 1B), which was higher than that of Met in either AD type (147 μmol/L) or carriers (80 μmol/L). During follow-up, increased Met levels were usually paralleled with elevated Met/Phe ratio (.94–44.7) and tHcy (13.0–65.5 μmol/L). Ten patients had plasma Met levels exceeding 800 μmol/L at least once during follow-up (Figure 1C) and then intervened with low-Met formula, diets appropriate for their ages without an excess of proteins, or drug administration. According to their latest evaluation, they had normal growth and development. A wider range of phenotypes were presented in these 10 patients, such as increased liver enzymes, verbal difficulty, motor delay, advanced bone age, developmental delay, and white matter lesions (Table 1). All these clinical symptoms were temporarily observed. Additionally, acidosis, hyperammonemia, or hypoglycemia were absent during follow-up, irrespective of infection. However, we also found asymptomatic cases harboring biallelic *MAT1A* variants. For example, P34, harboring novel compound heterozygous variants c.547C>G/p. Q183E and c.242G>A/p. R81Q had an initial Met 60 μmol/L that was below the cut-off value (81.7 μmol/L), a negative result according to biochemical indicators in NBS.

As MATD is a rare metabolic disorder with a limited population, detailed records of patients with neurological abnormalities are important evidence for better management of other cases in the future. Here, five patients with a Met value >800 μmol presented as follows: patients P16, P25, P27, and P31 had neurological abnormalities, and P35 carried 3 alleles with the same allele (c.695C>T/p. P232L) in P16.

**FIGURE 2:**
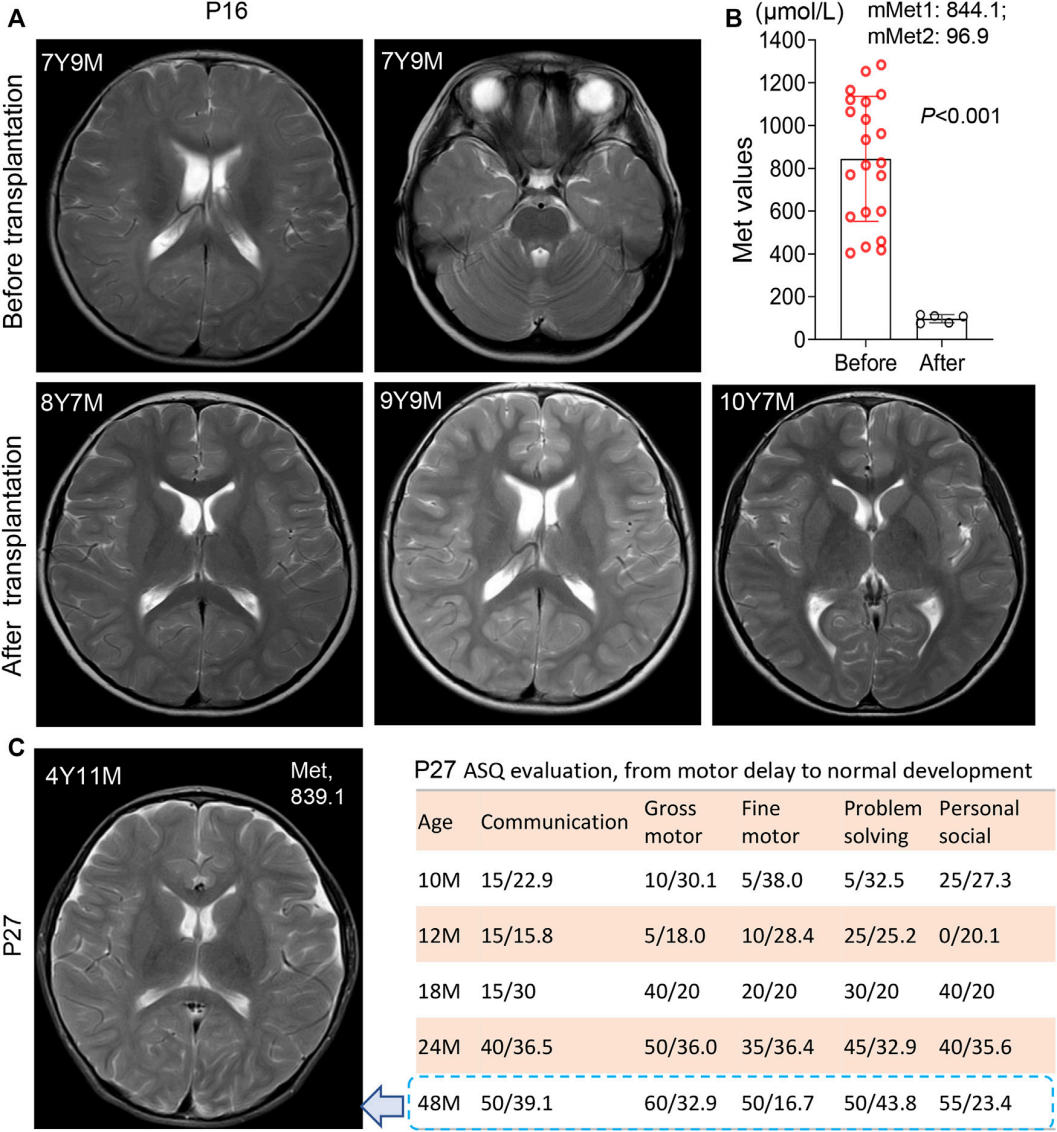
White matter lesions and development status in patient 16 and patient 27. **(A)** Brain MRI scanning before and after liver transplantation in patient 16 at different time points. 7Y9M = patient at 7 years-9 months old. Patient 16 harbored a heterozygosis c.695 C>T/p. P232L. White matter lesions was reversed after liver transplantation. **(B)** Significant decrease of Met levels after transplantation in patient 16, from 844.1 μmol/L (before) to 96.9 µmol/L (after). mMet1:844.1 = mean Met value before transplantation was 844.1 μmol/L; mMet2:96.9 = mean Met value after transplantation was 96.9 μmol/L **(C)** Brain MRI scanning of patient 27 at the age of 4 years 11 m with anomalies in white matter. The plasma Met concentration was maintained at 839.1 μmol/L left panel display his development status measured by ASQ (Ages-Stages Questionnaire). P27 had a normal development after SAMe supplementation. *, *p* < .05; **, *p* < .01; ***, *p* < .001.

Patient 16 clinically best fits with the autosomal recessive biallelic population that only one *de novo* variant c.695 C>T/p. P232L identified. Further evaluation of the intronic allele is needed. He had an initial Met 257 μmol/L, a high ratio of Met/Phe 4.58, and elevated plasma tHcy (59 μmol/L) with persistent hypermethioninaemia during follow-up. The recall Met was increased to 1283 μmol/L in the first month of life. Immediately, a diet with restricted protein and vitamin B6 was prescribed. At 2 months of age, the low-Met formula was added at a ratio of 5:1 to ordinary infant milk powder. The plasma Met levels during treatment were maintained at 403–599 μmol/L with a slight elevation of tHcy 17.6–19.0 μmol/L. At 20 months old, he had an intelligence score of 82 and an action score of 92 according to the Bailey Scale of Infant Development (BSID). Due to difficulties in persisting in a low-met restriction diet when grown, Met levels fluctuated within 1110–1165 μmol/L after 3 years of age. At age 7 years 9 m, brain MRI examination revealed a large range of abnormally high intensity in T2-weighted images with a reduced diffusion of white matter (Figure 2A), which has been observed in patients with phenylketonuria and glutaric aciduria type I (Manara et al., 2009; Tsai et al., 2017). We then were told that he had liver transplantation at the age of 8 years 1 m in another hospital. After transplantation, he picked up the normal diet and had a plasma Met of 74.2 μmol/L, 78.5 μmol/L, and 109.6 μmol/L at ages 8 years 4 m, 8 years 7 m, and 10 years 11 m, respectively. The mean plasma Met dramatically dropped from 844.1 μmol/L to 96.9 μmol/L (Figure 2B). Strikingly, the white matter lesions were gradually reversed to normal after the operation.

Patient 25 was homozygous for a novel variant c.875G>T/p. R292L with transient verbal difficulty and weakness in the right foot when walking. She had a remarkable increase in Met levels of 921 μmol/L when recalled for confirmation and then started on a mixture of low-Met formula and breastfeeding with betaine and folate. The Met value dropped to 599 μmol/L at 8 m of age. However, she could not adhere to the Met-restricted diet after 1 year of age. Fluctuated Met (628–850 μmol/L) values were observed with intermittent interventions. However, her development status was normal on the ASQ test, and both height and weight were P75 at follow-up at the age of 42 m.

Patient 27 was homozygous for a reported variant c.769G>A/p. G257R with persistent hypermethioninaemia. During the first 3 months of life, his Met values were persistent at 1069, 1097, and 1408 μmol/L, respectively, with a low-Met formula diet. At the age of 10 m, he had a motor delay with a gross motor score of 10 and a fine motor score of five on the ASQ evaluation. After SAMe supplementation and rehabilitation training, his ASQ score climbed to normal at 48 m of age, with a gross motor score of 60 and a body weight of P50. However, MRI showed mild hyperintense T2-weighted images in the white matter of cerebral hemispheres and widened bilateral frontotemporal extracerebral space (Figure 2C) at 4 years 11 m of age with a Met level of 839 μmol/L.

Patient 31 had compound heterozygous variants, c.769G>A/p. G257R and c.875G>T/p. R292L. With a Met-restricted diet, his Met was decreased from 977 μmol/L at age 1 m to 159 μmol/L at age 5 m and then manifested <500 mol/L. A transient development delay was observed at age 2 years, with a developmental quotient of 68 by the Gesell system, but disappeared at follow-up with a P50 score in both his height and weight at 5 years 10 m.

Patient 35 harbored three alleles, a paternal c.695C>T/p. P232L variant and two novel maternal variants c.608T>A/p. I203N and c.922G>C/p. A308P, with an initial Met of 183 mol/L. his Met value was 600 μmol/L and then increased to 808 μmol/L at the age of 2 m. Given low Met intake and betaine treatment, the Met fluctuated between 543-595 μmol/L until the age of 13 m, with normal growth and development. However, his Met increased up to 713–982 μmol/L after the protein restriction diet was discontinuous. Both his height and weight were P75 as of follow-up at the age of 108 m.

**FIGURE 3:**
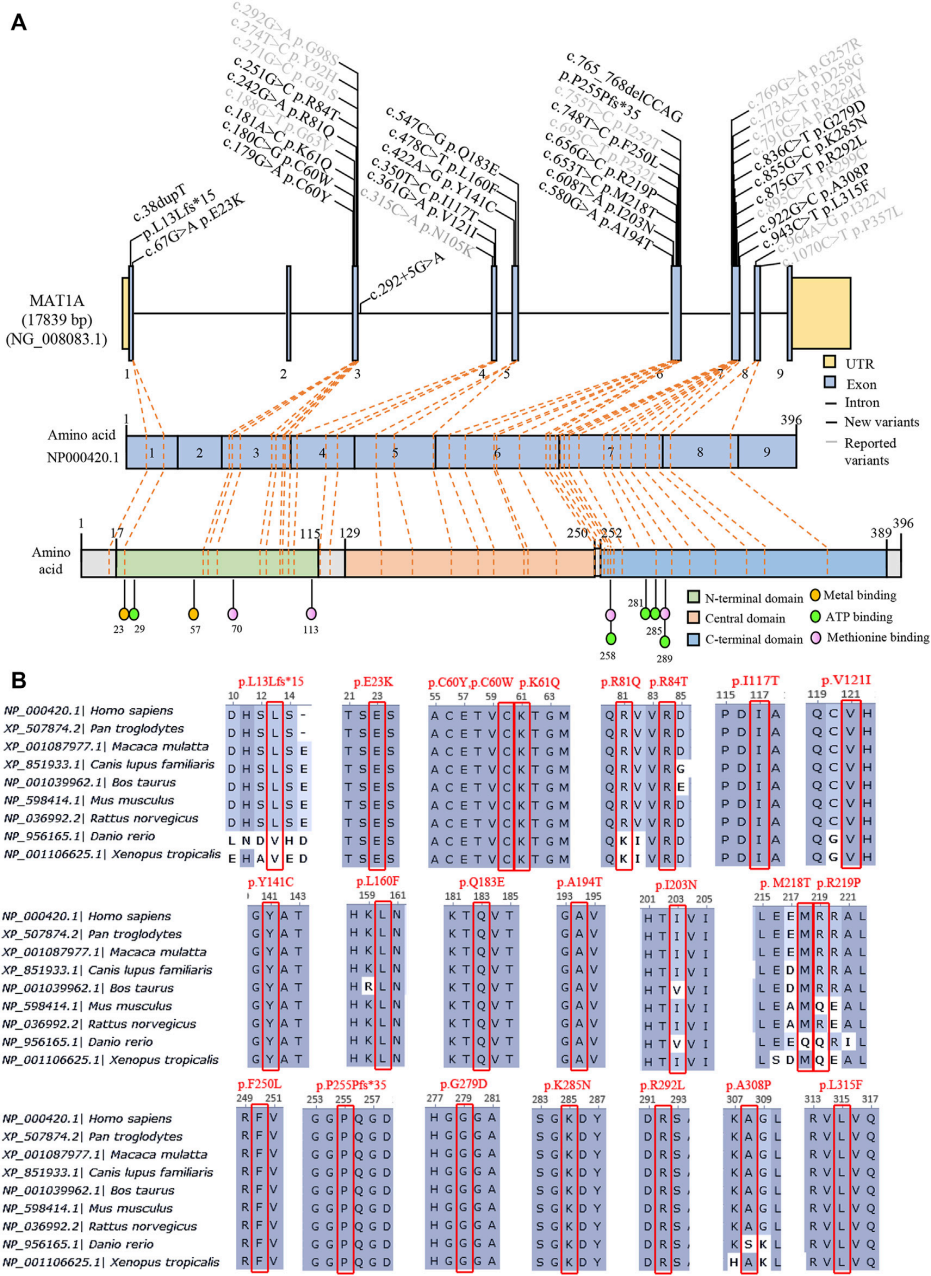
Summary of 38 variants of *MAT1A* identified in MATD patients and carriers. **(A)** Distribution of 38 variants in the *MAT1A* gene and protein among MATD patients and carriers. Black, 24 novel variants found in this study; grey, reported variants in MATD elsewhere. **(B)** Conservation analysis of 24 novel variants in different species, excluding the splicing c.292 + 5G>A.

Genetic characteristics in the AR type were more heterogeneous than the dominant type. Biallelic variants were genotyped in 18 patients, and three alleles occurred in P35. Sixteen patients harbored compound heterozygotes, while only 3 patients carried homozygotes, homozygous c.1070C>T/p. R357L in P22, homozygous c.875G>T/p. R292L in P25, and homozygous c.769G>A/p. G257R in P27 (Table 1). Surprisingly, each patient had a unique genotype. A total of 26 variants were identified, including 24 missense variants and two truncating variants (c.38dupT/p. L13Lfs*15 and c.765_768del CCAG/p. P255Pfs*35) and distributed randomly in genes and protein domains, except in exons 2 and 9 (Figure 3A). Notably, 17 novel variants in patients were found and predicted by automatic tools (Supplementary Table S2), of which c.242G>A/p. R81Q occurred in two asymptomatic individuals (P33 and P34), indicating a benign protein function as prediction *in silico*. However, the novel truncating variants were postulated to be pathogenic (P) or likely pathogenic (LP) according to ACMG recommendations (Richards et al., 2015). Most variants had high conservation in mammals (Figure 3B). Variants distributed in patients as c.188G>T/p. G63V in five individuals, c.895C>T/p. R299G in four individuals, c.769G>A, c.875G>T, and c.242G>A in two individuals each, and others appeared once.

### 31 MATD carriers

Thirty-one carriers (21 females and 10 males) were detected by the cutoff index in NBS, as shown in Table 2. Three carriers, C2, C14, and C25, were born prematurely. As shown in Figure 1, the initial Met values of each carrier were <100 μmol/L, ranging from 45 to 95 μmol/L with a mean value of 73 μmol/L. Six previously reported variants occurred in 18 individuals, distributed as c.1070C>T in eight individuals, c.769G>A in five individuals, c.773A>G in two individuals, and c.188G>T, c.271G>C and c.964A>G each in one child. Intriguingly, 8 novel variants were detected in 13 individuals, including one splicing and eight missense variants, of which c.181A>C/p.K61Q appeared in both carrier (C6) and patient (P20) as a recessive variant (Supplementary Table S2). As it is difficult to distinguish whether the novel variants were pathogenic in the dominant type or not, parents were advised to perform gene testing and Met assays to check whether parents harboring the same novel variant have a normal Met value. As shown in Table 2, parents’ Met values harboring novel variants ranged from 19-50 μmol/L with a low ratio of Met/Phe (<1.1) and were healthy, indicating those 6 novel variants, c.67G>A, c.181A>C, c.251G>C, c.292 + 5G>A, c.748T>C, c.748T>C, and c.855G>C, were transmitted as recessive type. Unfortunately, the parents harboring c.179G>A/p. C60Y or c.180C>G/p. C60W did not consent to the test. Cautiously, to avoid missing any AD cases, all carriers were followed up until the Met value was <50 μmol/L, or the Met value decreased continuously at the next three tests after NBS. Interestingly, most of the carriers had normal Met values <50 μmol/L by the age of 10 m, and their growth and development statuses were normal and healthy.

**TABLE 2:** Summary information of 31 carriers including Met values from one of parents.

Carriers/sex	NBS			Allele	From	Follow-up		Met/(Met/Phe) of father or mother
	Met (µmol/L)	Met/Phe	tHcy (µmol/L)			Mean (min-max) met (µmol/L)	Min-max met/Phe	
C1, M	95	1.7	10.7	c.67G>A/p.E23K	maternal	113.6 (40.5–213)	1.05–1.56	22/0.46
C2, F	84	0.85	16.3	c.179G>A/p.C60Y	maternal	61.8 (41–82)	0.83–1.79	NA
C3, F	98	1.69	13	c.179G>A/p.C60Y	NA	99.3 (89–110)	1.21–1.74	NA
C4,M	55.8	0.97	—	c.179G>A/p.C60Y	paternal	55.4 (49–61)	1.06–1.69	NA
C5, F	93	1.69	8.8	c.180C>G/p.C60W	paternal	51.5 (35–73)	0.72–2.03	NA
C6, F	55	1.11	19.6	c.181A>C/p.K61Q	maternal	62.2 (41–76.3)	1.11–1.48	27/—
C7, F	76	1.52	7	c.251G>C/p.R84T	maternal	61.7 (38–106)	1.01–1.96	19.32/—
C8, F	63	1.23	11.4	c.292 + 5G>A	paternal	66.5 (35–93)	0.76–1.74	25/—
C9, F	68	1.3	11	c.315C>A/p.N105K	paternal	88.9 (47–137)	0.75–1.35	42/0.54
C10, M	62	1.27	10.1	c.315C>A p.N105K	paternal	68.7 (24–118)	0.52–2.05	36/0.51
C11, F	55	0.98	—	c.315C>A p.N105K	maternal	91.6 (55–115)	1.25–2.4	NA
C12, F	84	1.62	10	c.748T>C/p.F250L	paternal	63.5 (31–98)	0.53–1.76	50/0.74
C13, F	51	1.29	12.8	c.855G>C/p.K285N	maternal	62.0 (51–75)	1.1–1.65	23/0.68
C14, F	86	0.97	10.3	c.188G>T/p.G63V	maternal	84.0 (61–95)	0.89–1.45	19/0.89
C15, M	36	0.64	—	c.271G>C/p.G91S	paternal	46.0 (29–70)	0.7–1.6	NA
C16, F	96	1.1	14.9	c.773A>G/p.D258G	maternal	54.7 (34–86)	0.91–2.0	49/0.75
C17, F	42	0.77	—	c.773A>G/p.D258G	maternal	54.2 (24–85)	0.54–1.94	20.37/0.33
C18, M	87	1.54	10.4	c.769G>A/p.G257R	NA	55.3 (34–104)	0.65–1.80	NA
C19, F	78	1.32	17.3	c.769G>A/p.G257R	paternal	81.0 (37–127)	0.65–2.38	35.88/0.51
C20, M	64	1.51	10.3	c.769G>A/p.G257R	paternal	72.3 (49–98)	0.87–1.45	55/0.78
C21, M	83	1.44	14.9	c.769G>A/p.G257R	paternal	94.1 (62–123)	1.49–2.85	37.36/0.67
C22, F	82	1.48	8.7	c.769G>A/p.G257R	maternal	97.7 (80–116)	1.08–2.09	37/0.9
C23, M	75	1.78	16.9	c. 964A>G/p.I322V	NA	142.8 (73–206)	2.24–3.83	NA
C24, F	61	1.2	9.5	c.1070C>T/p.P357L	maternal	69.3 (42–88)	1.24–1.88	26/0.51
C25, F	91	1.3	12	c.1070C>T/p.P357L	NA	73.2 (48–98)	0.99–2.11	NA
C26, F	80	1.58	10.7	c.1070C>T/p.P357L	maternal	84.9 (55–115)	0.87–1.56	49/0.76
C27, F	67	1.43	11.5	c.1070C>T/p.P357L	maternal	102 (43–196)	0.81–3.08	25/0.51
C28, F	89	1.12	7.5	c.1070C>T/p.P357L	paternal	75.4 (60–88)	1.15–1.24	39.70/0.76
C29, M	71	1.13	10	c.1070C>T/p.P357L	paternal	94.4 (66–120)	1.29–2.48	30/0.42
C30, F	68	1.14	14.6	c.1070C>T/p.P357L	NA	178 (85–280)	1.88–4.75	NA
C31, M	67	0.97	10.3	c.1070C>T/p.P357L	paternal	61.8 (38–87)	0.9–1.8	NA

NA, not available; F, female; M, male.

## Discussion

In this retrospective study, we described the phenotypes, genotypes, and long-term prognosis of 35 patients with MATD based on newborn screening, with an estimated incidence of 1:116,161, consistent with previous reports in Asia (Chien et al., 2005; Shi et al., 2012; Nagao et al., 2013). Newborn screening is non-substitutable to identify patients with severe MATD, which should be considered a routine metabolic disorder in the screening system in our country. The Met value is the best primary marker for both diagnosis and prognosis, though initial Met values in MATD patients are somewhat overlapped with carriers. Patients with plasma Met levels >800 μmol/L should be treated in a timely manner. However, no excessive intervention will be needed for patients with plasma Met levels <500 μmol/L during follow-up, while the careful follow-up to monitor the Met value and Met/Phe ratio regularly was the main parameter for optimal care. Our data also showed that AR patients had higher phenotypic and genetic heterogeneity than AD patients. Twenty AR cases have unique genotypes and presented a wide range of clinical abnormalities from asymptomatic to white matter lesions. Neurological abnormalities were reversed here by liver transplantation or by the determination of SAMe. Moreover, 24 novel variants were identified in patients and carriers, including two truncating alleles (c.38dupT/p.L13Lfs*15, c.765_768delCCAG/p.P255Pfs*35) and a benign one (c.242G>A/p.R81Q).

**FIGURE 4:**
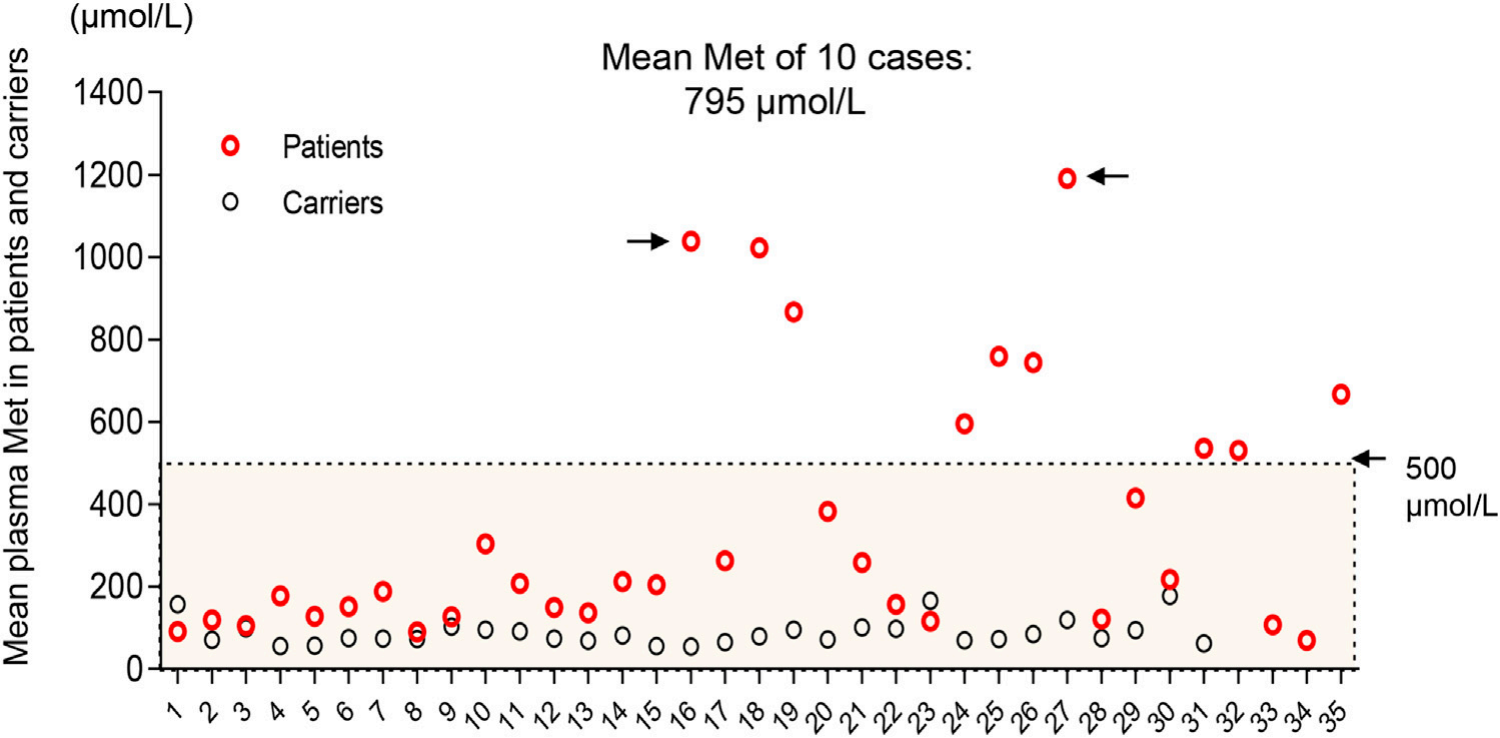
Distribution of mean plasma Met values in the first 4 months of life in patients (red) and carriers (black). Ten patients with interventions deviated from the other 25 cases with a mean Met >500 μmol/L. Black arrows indicate patients (P16 and P27) with white matter lesions.

Nevertheless, the Met value is still the best primary marker for both diagnosis and prognosis. The initial value of the Met and Met/Phe ratio is not correlated with neurological abnormalities in this MATD cohort. However, a significant difference was found in the initial Met levels between patients and carriers. Similarly, significant differences were presented in mean plasma Met values during follow-up among AD-type patients (147 μmol/L), AR-type patients (479 μmol/L), and carriers (80 μmol/L). The high level of plasma Met is correlated with the severity of the disease to a certain extent. Patients with plasma Met values >800 μmol/L had neurological abnormalities as shown in P16 and P27 as elsewhere (Mudd et al., 2003; Chien et al., 2015; Kido et al., 2019). Additionally, 24 patients with a mean value <300 μmol/L were clinically unaffected. Retrospectively, 10 individuals with interventions had higher fluctuations in plasma Met, as illustrated in Figure 1C. To limit the effect of treatment on the plasma Met value, we reappraised the mean Met values among patients and carriers based on the first 3 records by 4 months of age for comparison. The mean Met values in 10 therapeutic patients (795 μmol/L) were significantly different from those in the other 25 patients (Figure 4), indicating that the baseline value of Met for treatment was approximately 500 μmol/L. Patients P16 and P27 with white matter lesions had the highest mean plasma Met values of 1038 and 1191 μmol/L, respectively.

Methionine restriction was most useful for managing plasma Met values in our patients as well as SAMe supplementation (Hirabayashi et al., 2013). The Met-restricted diet was considered when plasma Met >500 μmol/L and was effective to decrease the plasma Met levels in AR patients here. Moreover, the Met value fluctuated to more than 800 μmol/L again when the protein restriction diet was discontinuous in patients P16, P25, and P35. Specifically, P27 was substantially unresponsive to a Met-restricted diet with persistent hypermethioninaemia and high Met levels >800 μmol/L by the age of 10 m. SAMe administration thereby was taken with a 400 mg/d dose and improved the development delay (Tada et al., 2004; Chien et al., 2015), with the gross motor score climbing from five at age 12 m to 60 at age 48 m. Although P27 was normal in physical and mental development, a mild hyperintense of T2-weighted images in white matter was still visualized in the brain MRI. Similar phenotypes were described in some older children with previous diagnoses of MATD (Chien et al., 2015). In this study, transplantation dramatically reversed the leukodystrophy situation, with a sheer decrease in mean plasma Met without dietary restriction from 844.1 μmol/L to 96.9 μmol/L in P16.

AR patients here had higher phenotypic and genetic heterogeneity than AD patients. The heterozygosity c.776C>T or c.791G>A surely caused no adverse long-range clinical effects on its patients (Chamberlin et al., 2000; Muriello et al., 2017). However, AR patients present a wide range of clinical symptoms, from asymptomatic to white matter lesions, which may partly be due to the complexity of genetic aetiology. More than 80 variants (Supplementary Table S1) have been reported according to available worldwide data since the molecular basis of the disease was established (Ubagai et al., 1995; Chamberlin et al., 1997). In the present study, we identified 38 variants distributed within patients and carriers, of which 24 were novel and predicted to be mostly damaged to protein function, except variant c.242G>A/p.R81Q, which has been previously classified as a variant of uncertain significance (variant reference 431706 In ClinVar). Two patients, P33 and P34, harboring the c.242G>A variant are asymptomatic, indicating that c.242G>A is benign. As each of the (McHugh et al., 2011) AR patients had unique genotypes, it was challenging to predict the relationship between clinical phenotype and genotype. P22 with a homozygous genotype [c.1070 C>T]; [c.1070 C>T] was clinically unaffected, implying that c.1070 C>T is benign. Alleles c.695C>T, c.875G>T, and c.769 G>A were supposed to be deleterious on protein function as patients P16, P25, P27, and P31 harbored one or two of them had neurological abnormalities. Patients (P19 and P32) carrying truncating variants had plasma Met >800 μmol/L more than once during follow-up. However, more cases were needed to evaluate the relationship between genotype and phenotype.

Here, our long-term prognostic data will be helpful for better understanding the natural history of MATD and optimal management for cases in the future. However, there were some limitations: 1) the detection of SAMe levels in the blood or cerebrospinal fluid (CSF) of patients was not performed; 2) whether the dose of the SAMe was dependent on weight or Met values was not clear; 3) limited by the small number of MATD patients and higher genetic heterogeneity, the relationship between phenotype and genotype is mostly undefined.

## Data Availability

The original contributions presented in the study are included in the article/Supplementary Material, further inquiries can be directed to the corresponding authors.
