# Supplementary material for: Long-term prognosis of 35 patients with methionine adenosyltransferase deficiency based on newborn screening in China
Source: Front Cell Dev Biol. 2023 Jan 10;10:1059680. doi: 10.3389/fcell.2022.1059680 (PMC9871361; doi:10.3389/fcell.2022.1059680)
Supplement: Supplementary file 1 [file DataSheet1.PDF]

Supplementary Table 1 Review of reported variants in *MAT1A* associated with MATD

mutant	AA change	Ref	mutant	AA change	Ref
c.65C>T	p.S22L	[1]	c.757G>A	p.G253R	[30, 31]
c.74 _75delTG	p.V25Gfs* 7	[3]	c.763C>T	p.P255S	[2]
c.100T>A	p.C34S	[34]	c.769G>A	p.G257R	[4]
c.110T>C	p.I37T	[5]	c.772G>C	p.D258H	[34]
c.113G>A	p.S38N	[6]	c.773A>G	p.D258G	[4]
c.125T>C	p.L42P	[1]	c.776C>T	p.A259V	[5, 7]
c.164C>A	p.A55D	[9]	c.790C>T	p. R264C	[8]
c.169G>A	p.E57K	[15]	c.791G>A	p.R264H	[6, 8, 10-14]
c.169+1G>A	Splicing	[16]	c.812A>G	p.Y271C	[35]
c.IVS2-2a>g	Splicing	[15]	c.822G>C	p.W274S	[16]
c.188G>T	p.G63V	[3]	c.823G>C	p.G275R	[16]
c.191T>A	p.M64K	[18]	827insG	p.K351*	[17]
c.205G>A	p.G69S	[8]	c.836G>T	p.G279V	[16]
c.255delCA	p.D92*	[6]	c.838G>A	p.G280R	[19]
c.271G>C	p.G91S	[5]	c.839G>T	p.G280V	[2]
c.274T>C	p.Y92H	[20]	c.856G>A	p.D286N	[16]
c.291G>C	p.K97A	[19]	c.862A>G	p.T288A	[14]
c.292G>A	p.G98S	[6]	c.867G>T	p.K289N	[8]
c.315C>A	p.N105K	[34]	c.870G>A	**p.V290V**	[9]
c.345delA	p.P115Pfs*23	[22]	c.874C>T	p.R292C	[21]
c.360C>T	p.K120K	[23]	c.882T>C	**p.A294A**	[22]
c.407G>T	p.G136V	[33]	c.890C>A	p.A297D	[4]
c.412A>G	p.M138V	[5]	c.895C>T	p.R299C	[4]
c.426T>C	**p.A142A**	[9]	c.896G>A	p.R299H	[4]
c.433G>A	p.E145K	[15]	c.914T>C	p.L305P	[9]
c.487C>T	p.R163W	[19]	c.966T>G	p.I322M	[6, 9, 24]
c.527T>A	p.L176Q	[16]	c.964A>G	p.I322V	[4]
c.529C>T	p.R177W	[5] [22]	c.1005C>T	p.W355*	[19]
c.533C>T	p.P177L	[25]	c.1006G>A	p.G366R	[6]
539insTG	p.T18*	[4, 17, 26]	c.1031A>C	p.E344A	[6]
c.572_592dup		[25]	1043delTG	p.H350*	[17]
c.589delC	p.P197Lfs*26	[18] [16]	c.1064T>G	p.L355R	[14]
c.595C>T	p.R199C	[14, 17]	c.1066C>T	p.R356W	[35]
c.596G>A	p.R199H	[16]	c.1067G>A	p.R356Q	[17]
c.623A>C	p.E208P	[34]	c.1067G>C	p. R356P	[8, 20]
c.656G>T	p.R219L	[5]	c.1067G>T	p.R356L	[21]
c.688G>A	p.V230M	[15]	c.1068C>T	p.R356W	[4]
c.689T>G	p.V230G	[16]	c.1070C>T	p. P357L	[8] [9]
c.695C>T	p.P232L	[3]	c.1081G>T	p.V361F	[27]
c.712G>A	p.E238K	[28]	c.1086-3C>G	Splicing	[31]
c.734_735delAG	p.Q245P fs*20	[15]	c.1131C>T	**p.Y377Y**	[9]
c.739A>G	p.S247R	[15]	c.1132G>A	P.G378S	[17, 29,32]
c.745C>T	p.R249W	[8]	c.1141G >A	p.G381R	[4]
c.746G>A	p.R249Q	[19]	c.1161G>A	p.W387*	[29]
c.755T>C	p.I252T	[4]	c.1188G>T	p.X396Yfs*69	[8]

.

1. Linnebank M, Lagler F, Muntau A, et al. Methionine adenosyltransferase (MAT) I/III deficiency with concurrent hyperhomocysteinaemia: Two novel cases. Journal of Inherited Metabolic Disease 2005; **28**(6):1167-1168.
2. Sen K, Felice M D, Bannick A, Colombo R, and Conway R L. Mild Persistent Isolated Hypermethioninemia Identified through Newborn Screening in Michigan. Journal of Pediatric Genetics 2019; **8**(2):54-57.
3. Zhang Z, Sun Y, Wang Y, Ma D, Cheng W, and Jiang T. Clinical analysis of methionine adenosyltransferase I / III deficiency in 5 confirmed cases in neonatal screening. Journal of Chinical Pediatrics 2019; **37**(12):889-892.
4. Fernandez-Irigoyen J, Santamaria E, Chien Y-H, et al. Enzymatic activity of methionine adenosyltransferase variants identified in patients with persistent hypermethioninemia. Molecular Genetics and Metabolism 2010; **101**(2-3):172-177.
5. Chadwick S, Fitzgerald K, Weiss B, and Ficicioglu C. Thirteen Patients with MAT1A Mutations Detected Through Newborn Screening: 13 Years' Experience. JIMD reports 2014; **14**: 71-6.
6. Chamberlin M E, Ubagai T, Mudd S H, et al. Methionine adenosyltransferase I/III deficiency: Novel mutations and clinical variations. American Journal of Human Genetics 2000; **66**(2): 347-355.
7. Muriello M J, Viall S, Bottiglieri T, Cusmano-Ozog K, and Ferreira C R. Confirmation that MAT1A p.Ala259Val mutation causes autosomal dominant hypermethioninemia. Molecular Genetics and Metabolism Reports 2017; **13**:9-12.
8. Chien Y H, Chiang S C, Huang A, and Hwu W L. Spectrum of hypermethioninemia in neonatal screening. Early Human Development 2005; **81**(6):529-533.
9. Ubagai T, Lei K J, Huang S, Mudd S H, Levy H L, and Chou J Y. Molecular Mechanisms of an Inborn Error of Methionine Pathway - Methionine Adenosyltransferase Deficiency. Journal of Clinical Investigation 1995; **96**(4):1943-1947.
10. Chamberlin M E, Ubagai T, Mudd S H, Levy H L, and Chou J Y. Dominant inheritance of isolated hypermethioninemia is associated with a mutation in the human methionine adenosyltransferase 1A gene. American Journal of Human Genetics 1997; **60**(3):540-546.
11. Martins E, Eusebio F, Marcao A, Rocha H, and Vilarinho L. Five families with hypermethioninemia associated with the dominantly inherited methionine adenosyltransferase I/III form deficiency. Journal of Inherited Metabolic Disease 2007; **30**: 6-6.
12. Couce M L, Boveda M D, Castineiras D E, et al. Hypermethioninaemia due to methionine adenosyltransferase I/III (MAT I/III) deficiency: Diagnosis in an expanded neonatal screening programme. Journal of Inherited Metabolic Disease 2008; **31**:S233-S239.
13. Martins E, Marcao A, Bandeira A, Fonseca H, Nogueira C, and Vilarinho L. Methionine Adenosyltransferase I/III Deficiency in Portugal: High Frequency of a Dominantly Inherited Form in a Small Area of Douro High Lands. JIMD reports 2012; **6**:107-12.
14. Couce M L, Dolores Boveda M, Garcia-Jimemez C, et al. Clinical and metabolic findings in patients with methionine adenosyltransferase I/III deficiency detected by newborn screening. Molecular Genetics and Metabolism 2013; **110**(3):218-221.
15. Nagao M, Tanaka T and Furujo M. Spectrum of mutations associated with methionine adenosyltransferase I/III deficiency among individuals identified during newborn screening in Japan. Molecular Genetics and Metabolism 2013; **110**(4):460-464.
16. Chien Y-H, Abdenur J E, Baronio F, et al. Mudd's disease (MAT I/III deficiency): a survey of data for MAT1A homozygotes and compound heterozygotes. Orphanet Journal of Rare Diseases 2015; **10**.
17. Chamberlin M E, Ubagai T, Mudd S H, Wilson W G, Leonard J V, and Chou J Y. Demyelination of the brain is associated with methionine adenosyltransferase I/III deficiency. Journal of Clinical Investigation 1996; **98**(4):1021-1027.
18. Hirabayashi K, Shiohara M, Yamada K, et al. Neurologically normal development of a patient with severe methionine adenosyltransferase I/III deficiency after continuing dietary methionine restriction. Gene 2013; **530**(1):104-108.
19. Kim Y-M, Kim J H, Choi J-H, et al. Determination of Autosomal Dominant or Recessive Methionine Adenosyltransferase I/III Deficiencies Based on Clinical and Molecular Studies. Molecular Medicine 2016; **22**:147-155.

20. Tada H, Takanashi J, Barkovich A J, Yamamoto S, and Kohno Y. Reversible white matter lesion in methionine adenosyltransferase I/III deficiency. American Journal of Neuroradiology 2004; **25**(10):1843-1845.

21. Furujo M, Kinoshita M, Nagao M, and Kubo T. S-adenosylmethionine treatment in methionine adenosyltransferase deficiency, a case report. Molecular Genetics and Metabolism 2012; **105**(3):516-518.

22. Sun Y, Ma D, Wang Y, Yang B, and Jiang T. Analysis of MAT1A gene mutations in a child affected with simple hypermethioninemia. Zhonghua yi xue yi chuan xue za zhi = Zhonghua yixue yichuanxue zazhi = Chinese journal of medical genetics 2017; **34**(1): 98-101.

23. Lin C, Zheng Q, Jiang M, and Lin Y. Newborn screening and variant analysis for methionine adenosyltransferase I/III deficiency. Zhonghua yi xue yi chuan xue za zhi = Zhonghua yixue yichuanxue zazhi = Chinese journal of medical genetics 2020; **37**(5): 527-531.

24. Mudd S H, Tangerman A, Stabler S P, et al. Maternal methionine adenosyltransferase I/III deficiency: Reproductive outcomes in a woman with four pregnancies. Journal of Inherited Metabolic Disease 2003; **26**(5):443-458.

25. Wang T, Ma J, Zhang Q, et al. Expanded Newborn Screening for Inborn Errors of Metabolism by Tandem Mass Spectrometry in Suzhou, China: Disease Spectrum, Prevalence, Genetic Characteristics in a Chinese Population. Frontiers in Genetics 2019; **10**.

26. Hazelwood S, Bernardini I, Shotelersuk V, et al. Normal brain myelination in a patient homozygous for a mutation that encodes a severely truncated methionine adenosyltransferase I/III. American Journal of Medical Genetics 1998; **75**(4):395-400.

27. Nashabat M, Al-Khenaizan S and Alfadhel M. Methionine adenosyltransferase I/III deficiency: beyond the central nervous system manifestations. Therapeutics and Clinical Risk Management 2018; **14**:225-229.

28. Ji Y, Nordgren K K S, Chai Y, et al. Human Liver Methionine Cycle: MAT1A and GNMT Gene Resequencing, Functional Genomics, and Hepatic Genotype-Phenotype Correlation. Drug Metabolism and Disposition 2012; **40**(10):1984-1992.

29. Kim S Z, Santamaria E, Jeong T E, et al. Methionine adenosyltransferase I/III deficiency: two Korean compound heterozygous siblings with a novel mutation. Journal of Inherited Metabolic Disease 2002; **25**(8):661-671.

30. Ma Y, Li D, Li X, et al. Hypermethioninemia caused by deficient activity of methionine adenosyltransferase. Journal of Chinical Pediatrics 2018; **36**(1): 57-60.

31. Nagao M, Tanaka T and Furujo M. Spectrum of mutations associated with methionine adenosyltransferase I/III deficiency among individuals identified during newborn screening in Japan. Mol Genet Metab 2013; **110**(4):460-4.

32. Hübner V, Hannibal L, Janzen N, Grünert SC, Freisinger P. Methionine Adenosyltransferase I/III Deficiency Detected by Newborn Screening. Genes (Basel). 2022;13(7):1163. doi: 10.3390/genes13071163.

33. Chamberlin ME, Ubagai T, Pao VY, Pearlstein RA, Yang Chou J. Structural requirements for catalysis and dimerization of human methionine adenosyltransferase I/III. Arch Biochem Biophys. 2000;373(1):56-62.

34. Zhao D, Ni M, Jia C,et al., Genomic analysis of 9 infants with hypermethioninemia by whole-exome sequencing among in Henan, China. Clin Chim Acta. 2022; 533:109-113.

35. Kido J, Sawada T, Momosaki K, Suzuki Y, Uetani H, Kitajima M, Mitsubuchi H, Nakamura K, Matsumoto S. Neonatal methionine adenosyltransferase I/III deficiency with abnormal signal intensity in the central tegmental tract. Brain Dev. 2019;41(4):382-388.

Supplementary Table 2 Functional prediction of 24 novel variants identified in patients and carriers

	mutant	AA change	Effect_refGene	SIFT_pred	Polyphen2_HVAR_pred	PROVEAN_pred	CADD_score	CADD_pred
Exon 1	**c.38dupT**	p.L13fs*15	frameshift	-	-	-	-	-
	c.67G>A	p.E23K	nonsynonymous	Damaging	Probably_damaging	Damaging	34	Damaging
Exon 3	c.179G>A	p.C60Y	nonsynonymous	Damaging	Possibly_damaging	Tolerable	27	Damaging
	c.180C>G	p.C60W	nonsynonymous	Damaging	Possibly_damaging	Tolerable	23.9	Damaging
	c.181A>C	p.K61Q	nonsynonymous	Damaging	Probably_damaging	Damaging	26.9	Damaging
	***c.242G>A**	p.R81Q	nonsynonymous	Tolerable	Benign	Tolerable	23.6	Damaging
	c.251G>C	p.R84T	nonsynonymous	Damaging	Probably_damaging	Damaging	29.9	Damaging
Intron 3	**#c.292+5G>A**		splicing					
Exon 4	c.350T>C	p.I117T	nonsynonymous	Damaging	Probably_damaging	Damaging	24.9	Damaging
	c.361G>A	p.V121I	nonsynonymous	Damaging	Probably_damaging	Tolerable	24.1	Damaging
Exon 5	c.422A>G	p.Y141C	nonsynonymous	Damaging	Probably_damaging	Damaging	26.1	Damaging
	c.478C>T	p.L160F	nonsynonymous	Damaging	Probably_damaging	Damaging	25.3	Damaging
	c.547C>G	p.Q183E	nonsynonymous	Damaging	Probably_damaging	Damaging	25.5	Damaging
Exon 6	c.580G>A	p.A194T	nonsynonymous	Tolerable	Benign	Damaging	23.3	Damaging
	c.608T>A	p.I203N	nonsynonymous	Damaging	Possibly_damaging	Damaging	31	Damaging
	c.653T>C	p.M218T	nonsynonymous	Damaging	Possibly_damaging	Damaging	26	Damaging
	c.656G>C	p.R219P	nonsynonymous	Damaging	Probably_damaging	Damaging	32	Damaging
	c.748T>C	p.F250L	nonsynonymous	Damaging	Probably_damaging	Damaging	24.7	Damaging
	**c.765_768delCCAG**	p.P255Pfs*35	frameshift					
Exon 7	c.836C>T	p.G279D	nonsynonymous	Damaging	Probably_damaging	Damaging	30	Damaging
	c.855G>C	p.K285N	nonsynonymous	Damaging	Probably_damaging	Damaging	29.2	Damaging
	c.875G>T	p.R292L	nonsynonymous	Damaging	Probably_damaging	Damaging	35	Damaging
	c.922G>C	p.A308P	nonsynonymous	Damaging	Probably_damaging	Damaging	28.2	Damaging
	c.943C>T	p.L315F	nonsynonymous	Damaging	Probably_damaging	Damaging	28.2	Damaging

#, c.292+5G>A was elevated by NetGene2-2.42 resulting in a confidence of 95% as a splicing mutant.

*, c.242G>A was predicted with a benign effect on protein function. Two patients harboring the mutant had no clinical phenotype. One patient of them had normal methionine concentration.
